# Artificial light at night disturbs the activity and energy allocation of the common toad during the breeding period

**DOI:** 10.1093/conphys/coz002

**Published:** 2019-02-06

**Authors:** Morgane Touzot, Loïc Teulier, Thierry Lengagne, Jean Secondi, Marc Théry, Paul-Antoine Libourel, Ludovic Guillard, Nathalie Mondy

**Affiliations:** 1 University of Lyon, Université Claude Bernard Lyon 1, CNRS, ENTPE, UMR5023 LEHNA, Villeurbanne, France; 2 Centre National de la Recherche Scientifique, Muséum National d’Histoire Naturelle (MNHN), UMR 7179, Brunoy, France; 3 Centre de Recherche en Neurosciences de Lyon (CRNL)—CNRS UMR 5292, Faculté de Médecine Laennec, Lyon Cedex, France

**Keywords:** Activity, amphibian, artificial light at night, energy balance, oxygen consumption

## Abstract

The presence of artificial light at night (ALAN) is currently a global phenomenon. By altering the photoperiod, ALAN may directly affect the physiology and behaviour of many organisms, such as the timing of daily rhythms, hormonal regulation, food intake, metabolism, migration and reproduction. Surprisingly while it is known that ALAN exposure strongly influences health of humans and laboratory animals, studies on wildlife remain scarce. Amphibians are one of the most nocturnal groups of vertebrates and exhibit an unfavourable conservation status in most parts of the world. In order to gain insight into the consequences of ALAN, we experimentally exposed 36 adult breeding male common toads, *Bufo bufo*, to a light intensity of 0.1, 5 or 20 lux for 20 days, to investigate the activity using infrared cameras and the whole-body oxygen consumption by respirometry, as well as body mass and food intake. ALAN reduced toad activity over 24 h by 56% at 5 lux and by 73% at 20 lux. It did not affect the total energy expenditure but altered energy allocation. Indeed, standard energy expenditure increased by 28% at 5 lux and by 58% at 20 lux, while activity energy expenditure decreased by 18% at 5 lux and 38% at 20 lux. Finally, body mass and food intake were not affected. This study suggests that ALAN plays a large role in the activity and energy metabolism of common toads, which may have a long-term negative effect on the fitness of common toad populations. Generalizing these results to other taxa is crucial for conservation of biodiversity in an increasingly light world.

## Teaser

Artificial nocturnal lighting, a worldwide phenomenon that is currently expanding, experimentally reduces the duration of activity and increases oxygen consumption at rest of the common toad, a nocturnal amphibian. These behavioural and physiological changes could be associated with an increase in individual stress levels and thus affect toad populations.

## Introduction

Among the anthropogenic pressures on biodiversity, artificial light at night (ALAN) dramatically expanded during the past century. In 2016, 23% of the Earth’s surface, 88% of Europe and almost half of the USA experienced brightness levels higher than natural thresholds (Falchi *et al.*, 2016). Recently, it was estimated that ALAN increased nearly 6% (ranging from 0 to 20%) per year over the last hundred years across the world (Hölker *et al.*, 2010a). This is due to the increase in urbanization and suburbanization (Grimm *et al.*, 2008), the high level of industrialization and the development of transport infrastructures (Gaston *et al.*, 2013) occurring during the last few decades. ALAN disturbs ecological processes by altering species abundance and distribution, and by modifying ecosystem functioning (Gaston *et al.*, 2014; Bennie *et al.*, 2015; Hölker *et al.*, 2015; Knop *et al.*, 2017). A wide diversity of organisms, such as insects, fishes, amphibians, birds, mammals and plants are affected by ALAN (Longcore and Rich, 2004; Gaston *et al.*, 2013). This is particularly the case of nocturnal species (Buchanan, 2006), representing 28% of vertebrates and more than 60% of invertebrates (Hölker *et al.*, 2010b). In these species, the duration, the level or the rhythmicity of nocturnal activity that determines key activities are performed at very low light level, such as foraging and reproduction. Hence, most of them may be impacted by an increase of light level due to ALAN (Buchanan, 1993; Tuxbury and Salmon, 2005; Polak *et al.*, 2011; Knop *et al.*, 2017). For example, in mouse lemurs, *Microcebus murinus*, exposed to nocturnal artificial light of 1.70 lux, the level of locomotor activity was significantly reduced compared to controls, while the duration of the nocturnal locomotor activity was not impacted (Le Tallec *et al.*, 2013).

Strong variations in activity level would likely affect energy balance regulation, and by extension, energy expenditure, food intake and body mass. Modifications of energy metabolism are widely recognized as a mediator of fitness (Auer *et al.*, 2015; Welbers *et al.*, 2017). When resources are limited, the energy available for an individual needs to be trade-off between competing traits, such as maintenance, survival and reproduction (Stearns, 1992), involving various behavioural and physiological processes (Welbers *et al.*, 2017). Several effects of lighting modification on energy expenditure have already been highlighted in birds (Welbers *et al.*, 2017), but also in amphibians. The spotted salamanders, *Ambrystoma maculatum*, kept at a 16:8 LD photoperiod had higher total oxygen consumption compared to salamanders kept at 8:16 LD photoperiod (Whitford and Hutchison, 1965). Hutchison and Kohl (1971) showed that the amount and the rhythm of oxygen consumption in the tropical toad, *Bufo marinus*, were altered under constant illumination (861 lux). However, the influence of artificial light on patterns of energy metabolism were generally not consistent between species, sexes and life-history stages. Responses to ALAN was also often depending on light intensities, spectral composition and exposure duration (Fonken *et al.*, 2010; Le Tallec *et al.*, 2013; Borniger *et al.*, 2014). Most of the time this information is not specified, which impairs inter-species comparisons. Moreover, the light intensities used in the experiments were often beyond the range of values to which organism are exposed to.

Areas restored or created for recreational purposes for local populations, as are many wetlands located in or around cities, may be particularly exposed to high levels of ALAN. A recent study measuring the intensity of nocturnal irradiance in a medium-size city in France showed that in urban and peri-urban wetlands close to the city, even if the light intensity is reduced by several orders of magnitude, the irradiance of artificial lighting underwater could reach or exceed irradiance levels as high as a full moon (Secondi *et al.*, 2017). Moreover, on overcast nights, irradiance exceeded full moon irradiance in half of the sites when measured above the water (Secondi *et al.*, 2017). Wetlands also host species whose populations have declined because of habitat loss and their conservation is important for local or regional diversity (Chester and Robson, 2013). These include amphibians, who have shown a significant decline in the last decades due to environmental disturbance (Wise, 2007). To our knowledge, few experimental studies explore in detail the physiological consequences of ALAN on amphibians (Wise, 2007; Perry *et al.*, 2008). Hence, it is of primary importance to determine how ALAN exposure alters key physiological parameters that may negatively affect their fitness and the dynamic of their populations. The common toad, *Bufo bufo*, an ubiquitous amphibian in Europe, is an explosive breeding species, and the short breeding season limits the number of pairing opportunities. Once they have emerged, males gather at breeding ponds in early spring, and females arrive either alone or already mated. Male–male competition takes place during the time interval between pairing and spawning. These energy-consuming behaviours—movement/displacement, foraging and reproduction—occur at night and may be influenced by ALAN. In addition, due to their high nocturnal visual sensitivity and their nocturnal activities, amphibians are expected to be affected by changes in brightness, such as ALAN (Buchanan, 2006).

In this context, we experimentally exposed toads to one of the three light intensities (0.1, 5 or 20 lux) during the entire breeding period. For each toad, we recorded the duration of activity over 24 h, measured food intake and energy expenditure, to determine energy balance and weighed individuals to highlight changes in body mass. Regarding the literature (Hutchison and Kohl, 1971; Turney and Hutchison, 1974; Mazerolle *et al.*, 2005; Vera *et al.*, 2005) and considering the nocturnality and the high sensitivity to nocturnal lighting of the common toad, we first expected ALAN to reduce their activity during night time and, as a result, to trigger modifications of the energy balance components. More specifically, we predicted a reduction of energy expenditure, resulting in the change of the energy balance.

## Materials and methods

### Animal collection and housing conditions

A total of 36 *B. bufo* males were captured in La Burbanche (45°50′48″N, 5°33′44″E) at the beginning of the breeding season (8 March 2017). This site, located 80 km north-east of Lyon (France), was chosen for its low level of ALAN (<0.01 lux regardless of weather conditions and lunar phase). Toads were collected at night by hand when patrolling the forest roads near a pond. Once brought back to the animal care facility (EcoAquatron, University of Lyon), toads were placed individually in large plastic boxes (47 × 36 × 25 cm^3^) containing 4 cm of litter, moistened twice a day. Ambient temperature and humidity were kept at 16.10 ± 0.90°C and 55.20 ± 2.90%, respectively, in each stall room. Throughout their captivity, toads were fed with live domestic crickets (*Acheta domesticus*), so that there were always six crickets in each box, a sufficient number of crickets so that toads were not restricted by food. After 2 days of acclimation in their respective box, toads were used for the experiments for 20 days and were released to their original site at the end of the experiments.

### Light treatments

The light/dark cycle was programmed by an electronic switch that was set to provide a 12:12 LD cycle. During the acclimation period, all toads were exposed during the day (start at 07:00) to the same light intensity of 426 ± 16 lux (neon Philips Master TL-D 58W/865) and during the night (start at 19:00) all toads remained under dark night conditions (<0.1 lux). After acclimation period, the toads were randomly assigned to one of the three light treatment groups (*N* = 12) and placed into three distinct rooms: a control group exposed to a light intensity of 0.10 lux (control group), corresponding to a typical full moon illuminance (Kyba *et al.*, 2017), a first experimental group exposed to a light intensity of 20 lux (20 lux-group), corresponding to the natural light intensity perceived in urban areas, such as public gardens (Gaston *et al.*, 2013) and a second experimental group exposed to an intermediate light intensity of 5 lux (5 lux-group), corresponding to the intensity experienced on a residential street (Gaston *et al.*, 2013). During the day (start at 07:00), toads were exposed to the same light intensity as during the acclimation period. During the night (start at 19:00), toads were exposed to their respective light treatment. To simulate ALAN, LED ribbons (LED white hot waterproof Light Plus SC-TWF-WW2) were placed on top of each box. A light diffuser and a yellow spectrum filter (Rosco Cinegel, #3152 Urban Vapour) were added on each LED ribbon to mimic sodium lamps (see Supplementary Fig. S1), which are the main urban nocturnal artificial lighting in France (Gaston *et al.*, 2013). Light intensities were measured at the bottom of the boxes with a lux meter (Illuminance meter T-10A, Konica Minolta, sensitivity threshold 0.01 lux) and checked every week (0.10 ± 0.01 lux for the control group, 22.89 ± 0.96 lux for the 20 lux-group and 5.70 ± 0.22 lux for the 5 lux-group).

### Activity

The activity of the 36 toads was recorded in the absence of crickets on the tenth day of light exposure (D10) in their respective boxes with their respective light treatment. The activity was defined as any movement of the body contributing, or not, to a position shift of the toad, independent of foraging. Toad activity was measured during 23 h ± 1 h with two infrared sensitive cameras (Point Grey Dragonfly2 DR2-HIBW) coupled with infrared LEDs (940 nm Vishay TSAL6400) invisible to the toads (Buchanan, 1993). Each camera was fixed on a tripod and recorded two boxes containing one toad each. This system allowed the cameras, connected to a computer, to record black and white pictures every 0.1 s. A real-time analysis of toad activity duration was estimated by calculating the number of pixels whose intensity changed more than 14 grey levels between two successive frames with the VPCore 2 software (Viewpoint SA). The duration of activity was automatically scored when above the threshold of 5 changing pixels/s. It was then expressed either in minutes over 22 h of measurement (between 10:30 and 08:30 the next day, hours between which we have recorded all the individuals), or in minutes over the daytime (between 10:20 and 19:00) or night time (between 19:00 and 08:30).

### Oxygen consumption

The oxygen consumption (VO_2_)—a proxy of the energy expenditure—was measured twice on 27 toads (nine randomly selected per group), during the first day of light exposure (D1, acute stress) and the last day of light exposure (D20, chronic stress). VO_2_ of each toad was measured during 23 h ± 1 h at the room ambient temperature (i.e. 16.10 ± 0.90°C). Effectively, each day, we had to move the recording systems according to the measured individuals, which explained the variation in the duration of the measurement. The day before and during the measurements, toads were fasting so that the nutritional status was the same for all animals and did not bias the VO_2_ measurement. Each toad was randomly placed in one of the four metabolic chambers, constituting the respirometer, and weighed at the beginning and end of the measurement. At the end of each measurement, all the chambers were carefully cleaned with water.

We used an intermittent-closed respirometer protocol (‘intermittent stop-flow system’), adapted from a method measuring fish metabolism (Lefevre *et al.*, 2011), using optodes (Firesting O2, Pyroscience GmbH, Germany), to continuously assess the oxygen level of air (%O_2_). The four translucent Plexiglas hermetic chambers of 539 ± 1 ml (9.5 × 9.5 × 5.9 cm^3^) constituting the respirometer did not constrain the toads and allow us to control the light intensity in the chambers to be the same as in their respective rooms, using the same LED ribbons as used previously. Solenoids, ordered by a Raspberry PI3 system (Raspberry PI3, UK), controlled the duration of the measurement (27 min) and the duration of the ‘flush’ (3 min), which composed each cycle (2 loops/h). The ‘flush’ period completely renews the chambers with fresh ambient air, continuously saturated in humidity with a pump (Alita Air Pump AL-60, debit: 60 l min^−1^), bubbling in a 2-l glass bottle filled with water (Lefevre *et al.*, 2011) to ensure that the air %O_2_ came back to a basal level. Furthermore, a peristaltic pump (Watson Marlow 205S, debit: 22 ml min^−1^) ensured continuous air circulation in the chambers to homogenize %O_2_ measurements. Air %O_2_ and temperature contained in the chamber were recorded every 5 s via the Oxygen Logger software (Pyroscience GmbH, Germany). Each individual VO_2_ measurement was calculated as the slope of the %O_2_ decrease during the measurement period.

For each measurement, one of the four chambers was randomly kept empty to control the proportion of %O_2_ variation not resulting from the toad (%O_2_ blank). %O_2_ blank values were subtracted from the %O_2_ measures, leading to %O_2_ corrected values, which allowed the calculation of VO_2_ of each individual in ml of O_2_ h^−1^ g^−1^ of body mass, following the formula:

$${\mathrm{VO}}_{2}=\frac{\frac{\Delta \%O_{2}}{\Delta t}\;\times \;60\times 60\;\times \;\left(Vb-\mathrm{Vc}\right)}{100\times \mathrm{Mb}}$$

where, Δ%O_2_/Δ*t* is the %O_2_ decrease in % of O_2_ s^−1^ during the measurement period, Vb is the chamber volume in ml, Vc is the toad volume in ml, considered equal to the toad body mass (volume density assumed to be equal to the mass density, 1 g = 1 ml) (Lefevre *et al.*, 2011), and Mb is the toad body mass in g. To consider the stress caused by toad handling at the beginning and end of VO_2_ measurement, the first 7 h and the last hour of the 23 ± 1 h of %O_2_ measurements were not integrated into the results.

**Figure 1:**
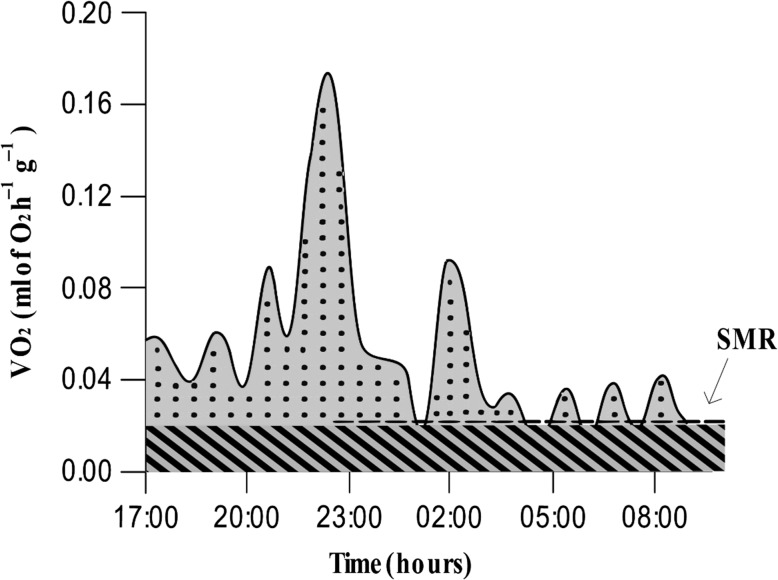
Toad VO_2_ illustrative trace. Standard metabolic rate (SMR), represented by the dotted line, is the mean of the lowest 10% of VO_2_ values (mean of the three lowest values here). Total energy expenditure, coloured in grey, is composed of the standard energy expenditure, represented by the hatching and the activity energy expenditure is represented by the dots.

VO_2_ measurements were used to calculate the total energy expenditure in ml of O_2_ g^−1^ of body mass for each individual, corresponding to the total area under the curve of VO_2_. For each individual, the standard metabolic rate (SMR, in ml of O_2_ h^−1^ g^−1^ of body mass) was calculated as the mean of the lowest 10% of VO_2_ values. SMR values are allowed to split total energy expenditure into two parameters: the standard energy expenditure, corresponding to the minimum energy used for the function and maintenance of the organism, and the activity energy expenditure. Those parameters were both expressed as a percentage of the total energy expenditure and determined as the area under the SMR and as the area under the curve of VO_2_ and above the SMR, respectively (Fig. 1).

### Body mass and food intake

Animals were weighed every 3 days, except for the days of VO_2_ measurements, where they were weighed for 2 consecutive days (LAB800-3000, B3C pesage, Sérénité). Body mass gain was expressed as a percentage of the initial body mass. Food intake in 24 h, in mg d^−1^ g^−1^ of body mass, was calculated as the difference between the given cricket mass and the remaining cricket mass. It was measured every 3 days, except for the days before and during the VO_2_ measurement and during activity measurements, when toads fasted.

### Data analysis

To investigate the effect of ALAN on total activity duration, food intake, and body mass gain, we used a linear model (LM) with light treatment (0.1, 5, 20 lux) as a fixed term. Normality and homoscedasticity criteria were not met for the duration of activity during the day and night; thus, we used a square root transformation on the data. We used a linear mixed model (LMM) to investigate how the duration of activity during day and night varied between light treatment and period. Light treatment (0.1, 5, 20 lux), period (day and night) and their interaction effect were introduced as explanatory terms in the fixed part of the model, and individuals were introduced as a random effect. To investigate how the SMR, total, standard and activity energy expenditure varied between light treatments and durations, we also used an LMM with light treatment (0.1, 5, 20 lux), duration of the experiment (D1 and D20) and their interaction as explanatory terms in the fixed part of the model and the individuals as a random effect. For the total energy expenditure, normality and homoscedasticity criteria were not met; thus, we used a logarithmic transformation on the data. Model selection was conducted by minimizing Akaike’s information criteria. A significance threshold of 0.05 was adopted for all the statistical analyses. All data were analysed using the statistical analysis software R 3.4.2 and the packages ‘nlme’, ‘car’, ‘lattice’, ‘MASS’, ‘stats’ (R development Core Team 2017).

### Ethical note

The capture of common toads was authorized by the Préfecture de l’Ain (DDPP01-16–145) and by the Ministère de l’Enseignement supérieur, de la Recherche et de l’Innovation (*APAFIS*#3655-2016011914372094) in accordance with the ethical committee of Lyon 1 University. No effect of transport on health or mortality was observed, and the housing conditions in the EcoAquatron housekeeping amphibians (University of Lyon) received the agreement of veterinary services (approval DSV 692661201).

## Results

### ALAN reduced toad activity duration

We detected activity for each individual in all light treatments. The total duration of activity of toads exposed to ALAN was significantly reduced after 10 days of exposure compared to the control (*F*_2, 15_ = 5.878, *P* = 0.013). Toads exposed to 5 and 20 lux decreased their mean total time spent moving by 56.7% (Tukey post hoc test *P* = 0.059) and 73.9% (Tukey post hoc test *P* = 0.013), respectively, compared to the control group.

**Figure 2:**
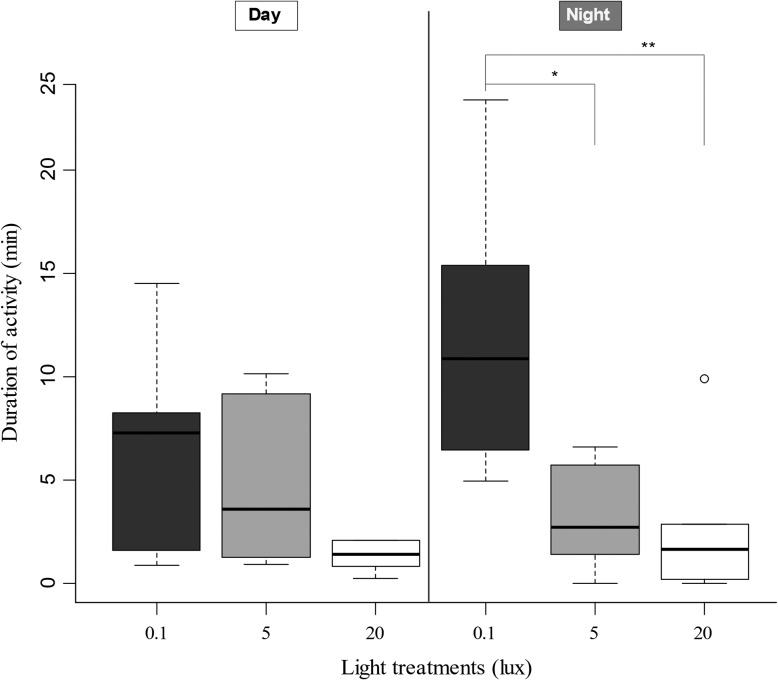
Day (left) and night (right) duration of activity (min) of toads after 10 days of exposure to 0.1, 5 or 20 lux (horizontal line: median value; box ends: upper and lower quartiles; whiskers: maximum and minimum values; dot: upper and lower outlier). Individuals were recorded in pairs for each light treatment; one value corresponds to two combined toads. Asterisks indicate statistical significance, * < 0.05 and ** < 0.01.

Furthermore, ALAN modified the time spent in activity during the day and at night differently (Fig. 2; lux*period: *X*^2^_2_ = 6.462, *P* = 0.040). While no difference was observed after 10 days of exposure during the day, toads exposed to ALAN significantly reduced their time spent in activity during the nocturnal period. Indeed, the mean duration of activity at night was reduced for the 5 lux-group and 20 lux-group by 73.4% (Tukey post hoc test *P* = 0.030) and 77.4% (Tukey post hoc test *P* = 0.009), respectively, relative to the control group.

### ALAN increased toad SMR, without impacting their total energy expenditure

**Figure 3:**
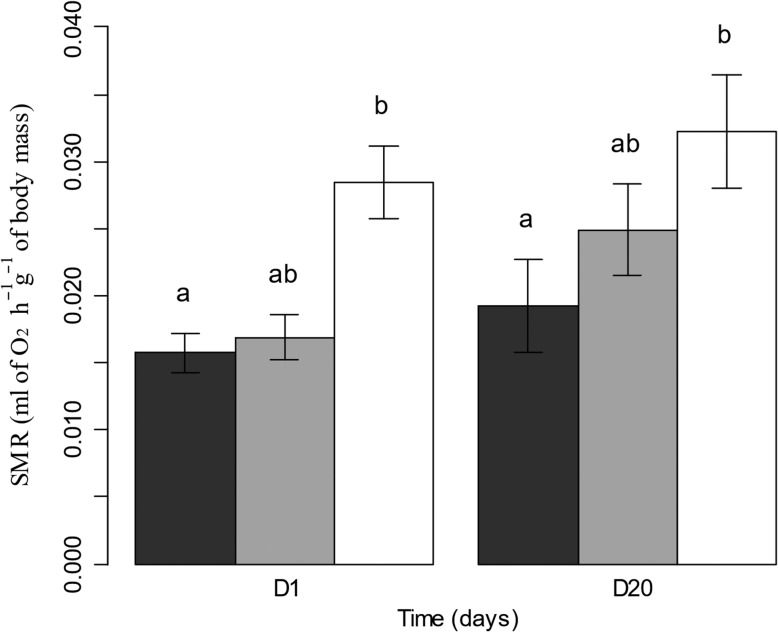
Standard metabolic rate (SMR) (ml of O_2_ h^−1^ g^−1^ of body mass) of toads exposed to 0.1 (black), 5 (grey) or 20 (white) lux after 1 (D1) and 20 (D20) days of exposure. *n* = 9 for each light treatment. Data are represented as the mean values ± SEM. Bars that do not share a letter are significantly different from each other.

The results indicated that regardless of the light treatment, toads spent the same amount of energy in 24 h, namely, 0.69 ± 0.04 ml of O_2_ g^−1^ of body mass on average. Indeed, the total energy expenditure in 24 h by toads was not altered either by the light treatment or by the duration of the exposure (lux: *X*^2^_2_ = 2.277, *P* = 0.320, period: *X*^2^_1_ = 2.294, *P* = 0.129). However, toad SMR was significantly increased by the light treatment (Fig. 3; *X*^2^_2_ lux = 18.353, *P* < 0.001). The SMR mean value in the control group was 0.0175 ml of O_2_ h^−1^ g^−1^ of body mass. The SMR increased on average by 18.4 and 74.2%, respectively, for the 5 lux-group and the 20 lux-group relative to the control. Even if the SMR increased with ALAN, only the 20 lux-group was significantly different from the control group (5 lux: Tukey post hoc test *P* = 0.436, 20 lux: Tukey post hoc test *P* < 0.001). Moreover, toad SMR was affected by the measurement period (period: *X*^2^_1_ = 6.145, *P* = 0.013), with an increase of 25% of the SMR mean value after 20 days of exposure compared to the very first hours of exposure, regardless light treatment.

### ALAN caused energy reallocation

**Figure 4:**
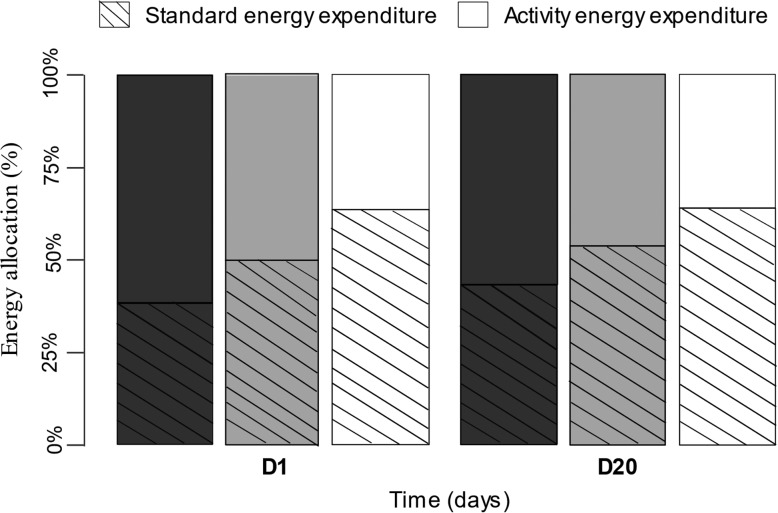
Mean standard (hatched bars) and activity (open bars) energy allocation (% of total energy expenditure) of toads exposed to 0.1 (black), 5 (grey) or 20 (white) lux at D1 and D20. *n* = 9 for each light treatment.

Standard energy expenditure and activity energy expenditure, the two components of the total energy expenditure, were affected by ALAN (Fig. 4). Control toads spent on average 0.25 ml of O_2_ g^−1^ of body mass in a resting state (standard energy expenditure) over 24 h, which represents 40.1% of their total energy over 24 h, and the remaining 59.9% (0.44 ml of O_2_ g^−1^ of body mass) was used for activity energy expenditure. The standard energy expenditure significantly differed between groups (lux: *X*^2^_2_ = 18.916, *P* < 0.001). Toads exposed to 5 and 20 lux increased their mean standard energy expenditure by 28.1% (5 lux: Tukey post hoc test *P* = 0.042) and 58.1% (20 lux: Tukey post hoc test *P* < 0.001), respectively, compared to the control group. In addition to the standard energy expenditure, the activity energy expenditure also significantly differed between groups. However, contrary to the standard energy expenditure, it decreased on average for the 5 lux-group and the 20 lux-group by 18.8 and 38.9%, respectively.

### ALAN impacted neither toad food intake nor toad body mass

**Figure 5:**
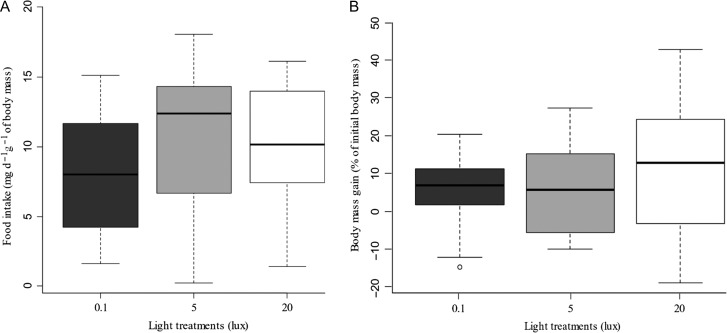
(**A**) Toad food intake (mg d^−1^ g^−1^ of body mass) after 20 days of exposure to 0.1, 5 or 20 lux. *n* = 12 for each light treatment. (**B**) Toad body mass gain (% of initial body mass) after 20 days of exposure to 0.1, 5 or 20 lux. *n* = 12 for each light treatment.

Exposure to ALAN did not modify the amount of crickets ingested by toads (Fig. 5A). After 20 days of exposure, we observed no significant differences between light treatments in food intake (*F*_2,33_ = 0.988, *P* = 0.383). Even if the body mass mean increased by 5.48% of the initial toad body mass in the control group, by 6.53% at 5 lux and 11.89% at 20 lux, there was no significant difference (Fig. 5B; *F*_*2*,33_ = 0.621, *P* = 0.543).

## Discussion

This study demonstrated that ALAN had a strong effect on the activity and energy allocation in the common toad. To our knowledge, no previous studies showed the consequences of exposure to nocturnal light intensities designed to mimic artificial light generated by street and outdoor lighting in peri-urban areas on amphibian energy balance.

We demonstrated that after 20 days of nocturnal exposure during the breeding period, at 5 or 20 lux, the total time spent in activity by male toads decreased by more than half. The reduction was almost exclusively due to the decrease in the time spent in activity during the night period. This response was expected, because a general decrease in activity under nocturnal illumination, due to modification of light intensity and/or light duration, has been documented in nocturnal species in numerous seminatural conditions and laboratory studies (Kramer and Birney, 2001; Vera *et al.*, 2005; Le Tallec *et al.*, 2013), and in the wild (Gaston *et al.*, 2013, Bennie *et al.*, 2014). Effectively, the effects observed in amphibians are similar to those observed in fishes and primates, which probably suggests a more or less universal pattern in nocturnal vertebrates. An alteration of the photoperiod by an exposure to light that ranged from 52 to 120 lux strongly reduced the circadian rhythm of locomotor activity in the green frog, *Rana clamitans* (Baker and Richardson, 2006). Elevated brightness at night (39 lux) for 30 s was demonstrated to reduce activity of American toads, *Bufo americanus*, by 62% relative to controls (Mazerolle *et al.*, 2005). In our experimental conditions, toads were recorded without evidence of predators or prey. Thus, the reduced activity seems to be explained by a direct response to ALAN via a behavioural and/or a physiological process (Kramer and Birney, 2001). Photoperiod is the major external synchronizer for many organisms, coordinating physiological and behavioural processes, which are modulated by an internal biological clock, to environmental events. The decrease in nocturnal activity in *B. bufo* experiencing ALAN illustrates the desynchronization of the activity pattern with the day/night cycle. This desynchronization can even go so far as to alter diurnal activity. In a study by Kramer and Birney (2001), Patagonian leaf-eared mice, *Phyllotis xanthopygus*, exposed to 1.5 and 3 lux showed a lower activity amplitude and shorter duration relative to controls but also an important and unexpected increase in diurnal activity, while individuals are strictly nocturnal. According to the authors, increased activity during the day could probably result from a decrease in nocturnal food consumption (Kramer and Birney, 2001). More broadly, such alterations of activity patterns could modify mechanisms related to moonlight, such as navigation (Longcore and Rich, 2004; Tuxbury and Salmon, 2005), habitat use (Clarke *et al.*, 1996), communication (Rand *et al.*, 1997; Baker and Richardson, 2006), reproductive behaviour (Rand *et al.*, 1997; Baker and Richardson, 2006), predation and foraging (Polak *et al.*, 2011; Russ *et al.*, 2014).

The reduced activity of exposed toads to ALAN could induce an alteration in the daily food intake. Contrary to our prediction, under our experimental conditions, 20 days of exposure did not alter the food intake over 24 h. Several laboratory studies showed that nocturnal light did not alter the total food intake over 24 h but triggered a disruption of the feeding rate rhythm. Grey treefrogs, *Hyla chrysoscelis*, exposed to white light of 3.8 lux, required more time to detect prey and to initiate the first prey capture attempt relative to control exposed to 0.003 lux (Buchanan, 1993). In contrast, Red-backed salamanders, *Plethodon cinereus*, exposed to a range of illumination (complete darkness to 10^−3^ lux) oriented toward prey sooner at higher ambient illumination, which could imply that prey detection is improved with increasing light levels (Perry *et al.*, 2008). Nevertheless, higher light levels delayed the nocturnal foraging activity of this species (Perry *et al.*, 2008). Furthermore, laboratory mice experimentally exposed for 8 weeks to 5 lux presented no difference in food intake in 24 h compared to controls but increased their percentage of food consumed during the light period (Fonken *et al.*, 2010) that contributes to an increase in their body mass and to metabolic alterations, such as impaired glucose tolerance. Here, we did not observe changes in body mass across treatments. Experiments in more restrictive nutritional conditions should be carried out to confirm those results.

In our experimental conditions, the total energy expenditure, estimated by measuring oxygen consumption, did not change during the experiment. Indeed, toads spent the same amount of energy in 24 h, whatever the light treatment. This result disagrees with the study on *P. major*, exposed to 7.6 lux of various spectra, for which the energy expenditure over 24 h dropped by 5–15% relative to birds exposed to dark (Welbers *et al.*, 2017). However, our result concerning total energy expenditure is consistent with the result we obtained regarding body mass. Indeed, the lack of variation of the total energy expenditure between light treatments associated with the non-variation of the food intake between light treatments reinforces the fact that body mass does not vary according to light treatments.

Several studies on amphibians have demonstrated the impact of an alteration of the photoperiod on oxygen consumption following exposure to strong artificial light intensities, but the results are so far inconsistent. In the tropical toad, *B. marinus*, exposed constantly to ~800 lux (consistent with daylight illumination), oxygen consumption no longer showed a circadian rhythm and the maximum oxygen consumption was reduced by 40% (Hutchison and Kohl, 1971). In the leopard frog, *Rana pipiens*, exposed continuously to artificial light, the 24-h oxygen consumption peak was maintained. However, it was lower and expressed with a 5-h time lag (Turney and Hutchison, 1974). In our study, where ALAN intensities are much lower, we also highlighted the alteration in toad oxygen consumption. We observed that energy was reallocated between activity (activity energy expenditure) and maintenance (standard energy expenditure), as individuals exposed to 5 and 20 lux reduced their activity energy expenditure by almost a fifth and more than a third, respectively, relative to the controls. The reduction of the activity energy expenditure seemed to be correlated with the decrease in the time spent active, as suggested by Hutchison and Kohl (1971). In addition, the reduction of the activity energy expenditure was equally observed from the first day of exposure and even after 20 days of exposure. This shows that toads did not acclimate to such ALAN levels at least over a 2-week period. It is important to note that earlier studies also have focused on the impact of photoperiod alterations on amphibian SMR following exposure to strong artificial light intensity. Hutchison and Kohl (1971) demonstrated a 27% SMR increase in *B. marinus* continually exposed to 861 lux, and this increase was 23% after 2 days of constant exposure to artificial light in *R. pipiens* (Turney and Hutchison, 1974). Our results, obtained with much lower ALAN intensities, are consistent with the literature. Individuals exposed to 5 or 20 lux increased their standard energy expenditure by more than a fifth or more than a half, respectively, compared to the controls. This increase was explained by the elevation of SMR in toads exposed to ALAN. A possible explanation for this SMR increase is the enhancement of the stress level of toads exposed to ALAN. Indeed, Wack *et al.* (2011) showed for the first time in amphibians that an increase in plasma corticosterone, the main stress hormone in amphibians (Troïanowski *et al.*, 2017), triggered an increase in the metabolism. One the other hand, several studies showed that anthropogenic disturbances, such as ALAN, could increase animal stress levels. In the same way, *P. major* adults breeding under 8.2 lux white ALAN showed higher baseline corticosterone levels compared to individuals breeding in dark conditions (Ouyang *et al.*, 2015).

All the effects we observed on male toads at the onset of the breeding period when they migrate towards breeding ponds and compete for mates suggest that increasing night brightness could be a serious threat that disrupts behaviour and physiology of many nocturnal amphibian species. The alteration of both activity and energy metabolism could impact the reproduction and ultimately lead to a reduction in fitness (Auer *et al.*, 2015). The physiological and behavioural effects of ALAN are currently overlooked in amphibians, and their ultimate consequences on the extinction probability of populations are not known. Our study showed that they should receive more attention and that ALAN should be considered as another risk factor when assessing the status of populations or species.

## Supplementary Material

Supplementary Data
