# Supplementary material for: Artificial light at night disturbs the activity and energy allocation of the common toad during the breeding period
Source: Conserv Physiol. 2019 Feb 6;7(1):coz002. doi: 10.1093/conphys/coz002 (PMC6364289; doi:10.1093/conphys/coz002)

Supplementary Figure S1

**Figure S1.** Irradiance spectrum of the experimental light source.

We used the lux meter (Illuminance meter T-10A, Konica Minolta, sensitivity threshold 0.01 lux) to set the experimental light intensities in our experimental boxes and we measured the irradiance of the LEDs with a spectroradiometer (JAZ, Ocean optics, Largo, FL, USA). The graph shows the relative irradiance spectrum of the LEDs used in this experiment. The light has a unique and narrow peak around 590 nm.


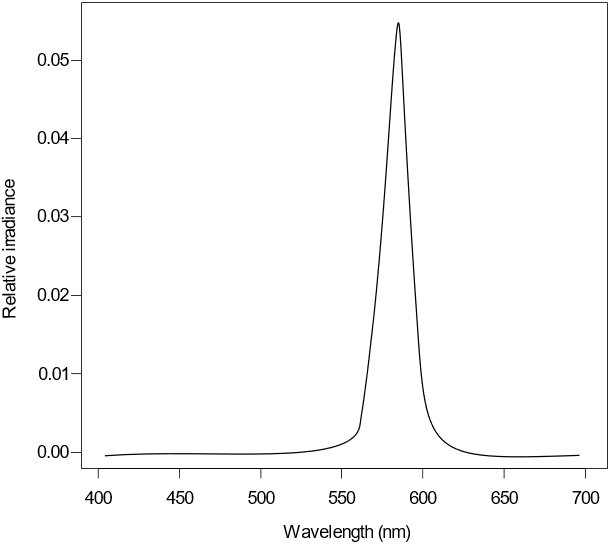

Supplement: Supplementary Data [file coz002_supplementary_figure_rev2.doc]
